# Supplementary material for: Use and Abuse of Electrocautery in Adenoidectomy Hemostasis
Source: Medicina (Kaunas). 2023 Apr 10;59(4):739. doi: 10.3390/medicina59040739 (PMC10145622; doi:10.3390/medicina59040739)
Supplement: Supplementary file 1 [file medicina-59-00739-s001.zip › medicina-2277512-supplementary.pdf]

CHILD’S AGE
SEX M / F
FOR HOW LONG DID THE POSTOPERATIVE PAIN LAST (IN DAYS)?
FOR HOW LONG HAVE YOU ADMINISTERED PAIN-KILLERS (IBUPROFEN, PARACETAMOL)? (IN DAYS)
FOR HOW LONG HAVE RINORRHEA / NASAL OBSTRUCTION LASTED? (IN DAYS)
HAS MALODEUR BEEN PRESENT IN THE POSTOPERATIVE DAYS? YES / NO
HAS EAR PAIN BEEN PRESENT? YES / NO
HAS NECK PAIN (POSTERIOR HEADACHE) BEEN PRESENT? YES / NO
HAS VELOPHARYNGEAL INSUFFICIENCY BEEN PRESENT IN THE FIRST POSTOPERATIVE DAYS? YES / NO

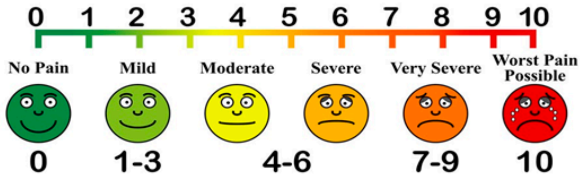

Figure S1. The questionnaire form designed for data collection from children’s parents in our study.
